# Controlling the Immunological Crosstalk during Conception and Pregnancy: HLA-G in Reproduction

**DOI:** 10.3389/fimmu.2014.00198

**Published:** 2014-05-13

**Authors:** Line Lynge Nilsson, Snezana Djurisic, Thomas Vauvert F. Hviid

**Affiliations:** ^1^Centre for Immune Regulation and Reproductive Immunology, Department of Clinical Biochemistry, Copenhagen University Hospital, Roskilde, Denmark

**Keywords:** MHC, HLA class Ib, HLA-G, human reproduction, pregnancy complications

## Abstract

In several years after its discovery in the placenta, the human leukocyte antigen (HLA) class Ib protein, HLA-G, was not given much attention, nor was it assigned great importance. As time has unraveled, HLA-G has proven to have distinctive functions and an unforeseen and possibly important role in reproduction. HLA-G is characterized mainly by its low polymorphism and restricted tissue distribution in non-pathological conditions. In fact, its expression pattern is primarily limited to extravillous cytotrophoblast cells at the maternal-fetal interface during pregnancy. Due to low polymorphism, almost the same protein is expressed by virtually all individuals. It is these unique features that make HLA-G differ from its highly polymorphic HLA class Ia counterparts, the HLA-A, -B, and -C molecules. Its function, seemingly diverse, is typically receptor-mediated, and involves interactions with a wide range of immune cells. As the expression of HLA-G primarily is limited to gestation, this has given rise to the hypothesis that HLA-G plays an important role in the immunological tolerance of the fetus by the mother. In keeping with this, it might not be surprising that polymorphisms in the *HLA-G* gene, and levels of HLA-G expression, have been linked to reproductive failure and pre-eclampsia. Based on recent studies, we speculate that HLA-G might be involved in mechanisms in reproductive immunology even before conception because HLA-G can be detected in the genital tract and in the blood of non-pregnant women, and is present in seminal fluid from men. In addition, HLA-G expression has been found in the pre-implanted embryo. Therefore, we propose that a combined contribution from the mother, the father, and the embryo/fetus is likely to be important. Furthermore, this review presents important aspects of HLA-G in relation to reproduction: from genetics to physiological effects, from pregnancy and pregnancy complications to a short discussion on future possible means of preventative measures and therapy.

## Introduction

The uterus and the placenta constitute a unique site of immune modulation where the semi-allogeneic fetus is tolerated by the maternal immune system. Both the mother and the fetus contribute to maintenance of tolerance. The mother through the presence of local regulatory immune cells that regulate redundant immune responses and the fetus, possibly among several mechanisms, through expression of non-classical human major histocompatibility complex (MHC) class Ib molecules, human leukocyte antigen (HLA)-E, -F, and -G, on extravillous trophoblast cells that infiltrate the decidua and make a direct contact with maternal immune cells (1–3). To the best of our knowledge, a classical antigen-presenting function, or capacity, of HLA-G has never been described, although the HLA-G molecule can bind peptides (4).

The expression of HLA-G is primarily limited to gestation and it has been widely studied in pregnancy because of its association with pregnancy complications, in particular pre-eclampsia and recurrent miscarriages (2, 5–8). The expression of HLA-G by embryos, as well as in the presence of soluble HLA-G (sHLA-G) in the maternal circulation, is associated with better pregnancy rates (9, 10). Furthermore, a different, possible role of HLA-G has been proposed in the context of remodeling of spiral arteries during placental development (11). However, further studies are needed to confirm this.

**Figure 1:**
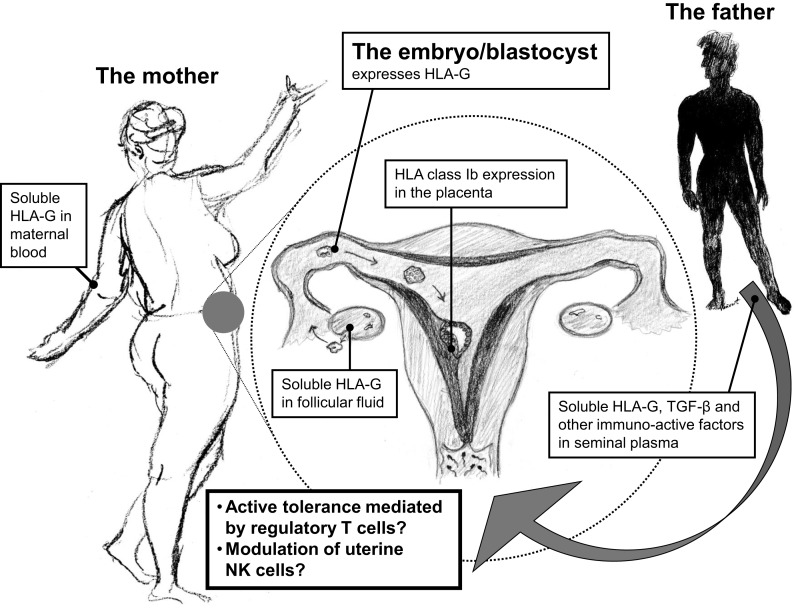
**An important and central role of HLA-G in reproduction may be depicted from its wide distribution within the reproductive cycle**. HLA-G is expressed in maternal blood, in follicular fluid, and in seminal plasma prior to implantation, and after fertilization in the blastocyst/embryo and in the placenta by the trophoblast cells. The continuous expression of HLA-G in the reproductive cycle may in particular modulate local immune cells in the female reproductive system for immunological acceptance of the semi-allogenic embryo.

An accumulating body of evidence suggests that HLA-G may be an important factor in reproduction even before conception. sHLA-G circulates in the blood of non-pregnant women (and in the blood of male donors), HLA-G is expressed in the female genital tract, and sHLA-G has been identified in seminal plasma (Figure 1) (12–16). In addition, HLA-G expression has been found in the pre-implanted embryo. Thereby, a combined contribution from the mother, the father, and the embryo, or fetus, is likely to be important. The aim of the present review is to give an overview of, and to discuss, important aspects of HLA-G in relation to reproduction: from genetics to physiological effects, from pregnancy and pregnancy complications to a short discussion on future possible means of preventative measures and therapy.

## The *HLA-G* Gene

### *HLA-G* polymorphisms in coding regions

**Figure 2:**
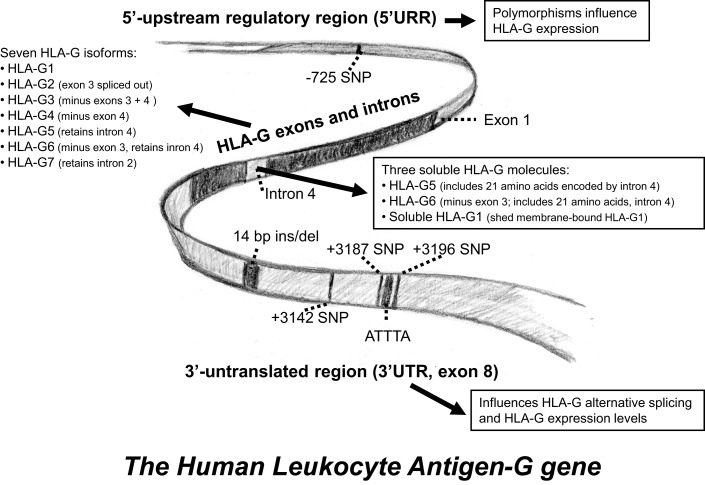
**A schematic representation of the *HLA-G* gene**. Some of the most intensively studied gene polymorphisms in the 5′-upstream regulatory region and in the 3′-untranslated region. An overview of the different HLA-G isoforms is included.

The *HLA-G* gene contains eight exons and seven introns (Figure 2). The external part of the HLA-G molecule consists of three parts, the α1, α2, and α3 domains (exons 2–4). The HLA-G full-length membrane protein is anchored in the cell membrane by the transmembrane region (exon 5). The cytoplasmic domain is encoded by exon 6 and the very first, short part of exon 8 (3, 17). Polymorphisms in the coding region of *HLA-G* are relatively scarce but evenly distributed between exons 2, 3, and 4, as well as in introns (6, 7, 18–20). Most polymorphisms do not alter the protein sequence, and the ones that do, allow for a grouping in major allele groups: G*01:01:xx:xx, G*01:01:xx, G*01:02, G*01:03:xx:xx, G*01:04:xx, G*01:05N (null allele), G*01:06, and G*01:07 to G*01:18. However, polymorphisms that define these allele groups have probably no effect on the secondary structures of the heavy chains, and the functional relevance of this nucleotide variability remains unclear. In total, 50 alleles and 16 allele groups representing an amino acid substitution have been described in the *HLA-G* gene sequence [WHO Nomenclature Committee for Factors of the HLA System and the International Immunogenetics Information System (IMGT)/HLA Database].

The polymorphic deletion of the first basepair (bp) of codon 130 or the third of codon 129, which results in a frameshift, defines the null allele (G*01:05N), and this null allele does not encode functional full-length HLA-G protein isoforms (HLA-G1 and -G5, see below) (18, 21). Nonetheless, studies show that these isoforms are not essential for fetal survival, indicating that expression of other HLA-G isoforms or HLA-E and/or -F, which are also involved in immune modulation in the placenta, may compensate for the lack of HLA-G1 and -G5 protein (22–24).

### *HLA-G* polymorphisms in non-coding regions

The functional mRNA level of *HLA-G* is governed by the rate of synthesis, mainly driven by the promoter region, or 5′-upstream regulatory region (5′-URR), as well as by the rate of degradation, stability, localization, and translation of the mRNA (25) (Figure 2). The rate at which pre-mRNA is produced is partly mediated by pre-transcriptional events, e.g., binding of transcription factors to regulatory motifs in the promoter region. HLA class I promoters sequences are generally conserved, but the *HLA-G* promoter is somewhat unique. Although its nucleotide sequence and structure is similar to other class I genes in several aspects, peculiarly, most regulatory motifs in the HLA-G promoter region are non-functional (20). Of importance, two main regulatory modules are flawed. First, the interferon (IFN)/Enhancer A region is blemished by a 16-bp deletion (17, 26), and second, the SXY module that mounts the transcriptional apparatus, represents a divergent sequence that does not allow for appropriate binding of the class II transactivator (CIITA) (27).

The 3′-untranslated region (3′UTR) of the *HLA-G* gene also exhibits several regulatory elements including AU-rich motifs and a poly-A signal to influence mRNA stability, turnover, mobility, and splicing pattern (20, 28). Polymorphisms in these regions may thus affect the expression of HLA-G by altered regulation of gene transcription or by destabilizing the mRNA transcript (28–30). Indeed, several important polymorphisms have been described in the 5′URR and the 3′UTR of the *HLA-G* gene (28, 29, 31–34).

### The 5′-upstream regulatory region of the *HLA-G* gene

Polymorphisms in the HLA-G 5′URR are close to regulatory elements and CpG sites, and are likely to alter binding of transcription factors and/or promoter methylation, and as a consequence influence the rate of transcription. Although sequence variation affecting transcription would be expected inside regulatory elements, most variable sites are not found in known motifs (20). Interestingly, an accumulating body of evidence indicates a balancing selection on the *HLA-G* promoter, and thereby indicates a preference for heterozygosity in which, possibly, individuals with both high- and low-expressing promoters are privileged (19, 29, 33, 35). Few studies address the direct association between HLA-G promoter SNPs and a differential HLA-G expression. One variation, a SNP at position −725 (rs123334), is associated with sporadic miscarriages and differential HLA-G expression (32, 36), and others, SNPs at position −1305, −964, and −486, are associated with yet other conditions like vitiligo, asthma, and acute allograft rejection in end-stage renal disease (37–39).

### The 3′ untranslated region of the *HLA-G* gene

In contrast to the coding region, the 3′UTR of the *HLA-G* locus presents a rather high degree of variation. Since the 3′UTR of the *HLA-G* gene exhibits several regulatory elements including AU-rich motifs, a poly-A signal, as well as signals that regulate the spatial and temporal expression of mRNA, the polymorphic sites may influence mRNA stability, turnover, mobility, and splicing pattern (20, 28, 30). A 14-bp ins/del (rs66554220) located in exon 8 is the best studied polymorphism in the 3′UTR, and has been shown to influence *HLA-G* mRNA transcript size and stability (19, 28, 30, 31, 40–42). The presence of the 14-bp-insertion sequence introduces an alternative splice site that generates a 92-bp deletion in the 3′UTR of the *HLA-G* mRNAs, and this alternative splice form seems to have an impact on the expression levels of HLA-G (5, 28, 40, 41). The positions of polymorphism in the 3′UTR of the *HLA-G* gene are in the current review numbered according to the publication by Castelli et al., which includes the 14-bp sequence in the reference sequence (43). At least three other SNPs in the 3′UTR are associated with *HLA-G* mRNA regulation and differences in sHLA-G levels: one positioned at +3142 (rs1063320) substituting a C to a G, another at +3187 (rs9380142) substituting an A to a G, and the third at position +3196 (rs1610696) where a C is substituted with a G (Figure 2). Studies show that polymorphisms in the 3′UTR probably act as targets for microRNAs, thereby controlling *HLA-G* mRNA stability and expression levels (8, 43–47). Furthermore, the +3187 and the +3196 SNPs are located just before and after an AUUUA motif associated with mRNA stability (28, 43).

### Combined 5′URR and 3′UTR *HLA-G* haplotypes

In some cases, polymorphism in the *HLA-G* 5′URR/promoter region may be in linkage disequilibrium with *HLA-G* 3′UTR variants (19, 33, 37), and some of them might influence alternative splicing and mRNA stability (30, 48). The −725 SNP located in the 5′URR is possibly in linkage disequilibrium with the 14-bp ins/del, the +3142, and the +3187 polymorphic sites, and is suggested to influence the stability of mRNA transcripts (36). Recently, the full combinations of 5′URR haplotypes, *HLA-G* WHO nomenclature alleles, and 3′UTR haplotypes in the HLA-G gene have been elucidated in a Brazilian population (33). The DNA polymorphisms in these extended *HLA-G* haplotypes may influence *HLA-G* expression and the stability of *HLA-G* mRNA transcripts in combination. Investigating the 5′URR and 3′UTR *HLA-G* extended haplotypes instead of evaluating single polymorphisms could determine the significance of allelic variants of *HLA-G* more accurately (33). A study correlating 3′UTR extended haplotypes with HLA-G soluble levels in a Brazilian and French cohort, showed that some haplotypes were associated with high sHLA-G levels (named UTR-1) and some with low sHLA-G levels in blood plasma from healthy donors (named UTR-5 and UTR-7) (47). However, full consensus does not exist in these studies, as another French study reported conflicting results (46). These different *HLA-G* haplotypes differ at the 14-bp ins/del, the +3142, the +3187, and the +3196 polymorphic sites in the 3′UTR, as well as in polymorphic sites in the 5′URR.

### Unique characteristics of HLA-G

A characteristic unique to HLA-G is the post-transcriptional alternative splicing of the mRNA from the single *HLA-G* gene. HLA-G1 represents the full-length isoform, whereas the other isoforms are formed by out-splicing of exons. This result in seven isoforms, four of which are membrane-bound (HLA-G1, HLA-G2, HLA-G3, and HLA-G4), and three of which are soluble (sHLA-G5, sHLA-G6, and sHLA-G7) (40, 49, 50) (Figure 2). HLA-G1 and HLA-G5 are the most studied isoforms. In contrast to other *HLA class I* genes, exon 6 of the *HLA-G* gene encodes a pre-mature stop codon that results in a truncated cytoplasmic tail (17). The truncated cytoplasmic tail results in a reduced endocytosis and thus a low cell surface turnover of the HLA-G molecule (51, 52). The soluble isoforms lack the transmembrane region due to a stop codon in intron 4 (40, 50). In the presence of metalloproteinases, HLA-G1 loaded with peptide can be shed from the surface by proteolytic cleavage, also resulting in a soluble molecule. HLA-G1 and sHLA-G5 are both capable of forming heterodimers with β2-microglobulin (β2m) (53).

## HLA-G Expression and Function in Relation to Reproduction

We propose that HLA-G might be involved in mechanisms in reproduction even before conception because HLA-G can be detected in the genital tract and in the blood of non-pregnant women, and is present in seminal fluid from men.

**Figure 3:**
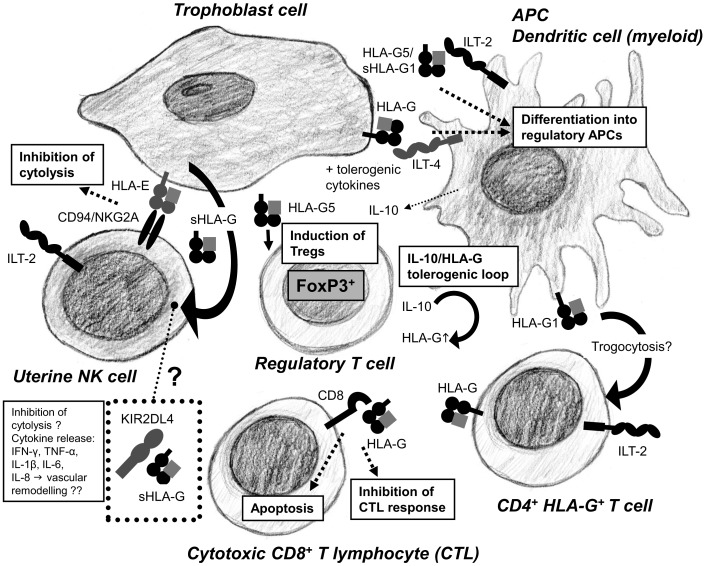
**Human leukocyte antigen-G is a key mediator of the tolerogenic loop arising from the crosstalk between immune cells in the placenta**. HLA-G promotes differentiation of DCs into tolerogenic DCs secreting IL-10, TGF-β, and expressing HLA-G. IL-10 and TGF-β induce Tregs. Tregs stimulate trophoblast cells to further upregulate expression of HLA-G. HLA-G can be acquired by CD4^+^ T cells through trogocytosis, increasing the pool of regulatory immune cells in the placenta. Cytotoxic CD8^+^ T cells are inactivated and undergo apoptosis by binding of HLA-G to the CD8 co-receptor. HLA-E presents HLA-G derived signal peptides and bind to the CD94/NKG2A receptors on uterine NK cells inhibiting cytotoxicity. Soluble HLA-G from trophoblast cells accumulates in KIR2DL4^+^ endosomes in uterine NK cells, which may result in active secretion of proangiogenic and proinflammatory cytokines, although this is controversial.

The function of HLA-G seems to be diverse, involving interactions with NK cells, cytotoxic T lymphocytes, regulatory T cells (Tregs), and it may be involved in regulating angiogenesis and cell migration (Figure 3). HLA-G is expressed by the extravillous trophoblast cells in the placenta, where the molecule has been attributed an important role in early placentation and maintenance of successful pregnancy (2, 3). Also, HLA-G is expressed in tissues important for the reproductive cycle. HLA-G is expressed in the follicular fluid and in the genital tract (15, 54, 55). Furthermore, HLA-G and sHLA-G has been detected in the male reproductive system and in semen (14, 56). After fertilization, HLA-G is expressed by the blastocyst and the early embryo (57, 58). HLA-G is also expressed at other immune privileged sites such as the cornea and thymic epithelial cells (59, 60). Finally, sHLA-G can be measured in peripheral blood from healthy female and male donors, and during pregnancy the concentration raises two to five times compared to what is observed in non-pregnant women (9, 12, 61, 62). One of the sources of soluble HLA-G5 in the blood of non-pregnant women and in men is most likely monocytes; though CD4^+^ and CD8^+^ T cells and B cells seem to be able to secrete HLA-G5 as well, although in lower amounts (63). However, during pregnancy a substantial amount of sHLA-G may be derived from the placenta by shed HLA-G1 and possibly secreted HLA-G5. sHLA-G levels in blood are associated with *HLA-G* gene polymorphisms and *HLA-G* haplotypes (19, 64).

The blastocyst implants into the uterine wall and the fetal-derived extravillous trophoblast cells invade the decidua and are involved in the remodeling of the spiral arteries. The maternal blood flow and tissue leukocytes are hence in direct contact with fetal trophoblast cells that express HLA-G (2, 65).

Still, it seems to be controversial, whether or not the soluble HLA-G5 and -G6 isoforms are secreted by trophoblast cells (66). Nonetheless, first trimester trophoblast expresses HLA-G5 and -G6 mRNA transcripts (28). One study has suggested that the major sHLA-G isoform in maternal serum should be HLA-G6 (or soluble HLA-G2) only consisting of heavy chains (67). However, to our knowledge these results have not been reproduced.

### Regulation of HLA-G expression

The regulation of HLA-G expression has been studied on different levels, and the majority of studies have focused on the importance of the genetic polymorphisms in the *HLA-G* gene as previously mentioned. Especially the 14-bp ins/del polymorphism in the 3′UTR, exon 8, of the *HLA-G* gene has been implicated in mRNA stability and hence the overall HLA-G production. However, conflicting reports on whether the 14-bp-insertion allele is associated with high or low level of expression of HLA-G exist. Experiments with *HLA-G* transductants showed that K562 cells with the 3′UTR 14 bp-insertion sequence had more stable mRNA compared to transductants lacking the 14-bp insertion. The study also looked at the functional impact of the 14-bp insertion on NK cell cytotoxicity, and found that K562 transductant cells carrying the 14-bp-insertion sequence were significantly less sensitive to NK cell cytotoxicity as compared to K562 cells that did not carry the 14-bp-insertion sequence (42). However, this study only investigated the isolated role of the 14-bp sequence in experiments performed with the use of a cell line, and the importance of the 14-bp-insertion sequence may be modulated or influenced by other linked polymorphisms in the 3′UTR, and in future studies it is important to study different *HLA-G* haplotypes. A study by Martelli-Palomino et al. showed that the 14-bp deletion allele correlated with higher blood plasma sHLA-G levels compared to the 14-bp insertion in extended 3′UTR *HLA-G* haplotypes (47). This is in line with previous studies, which have shown that the 14-bp ins/14 bp ins HLA-G genotype is associated with lower blood plasma and serum sHLA-G levels, compared to the 14-bp del/14 bp ins genotype and the 14-bp del/14 bp del genotype (12, 13). MicroRNAs have also been shown to regulate HLA-G expression and thereby function. In a study by Manaster et al., two microRNAs, miR-148a and miR-152 were shown to down-regulate HLA-G expression and thereby reduce the binding of HLA-G to its cognate inhibitory receptor immunoglobulin-like transcript 2 (ILT-2). And interestingly, in the placenta, the cellular content of miR-148a and miR-152 shown to reduce HLA-G expression was very low compared to other tissues. Therefore, it can be speculated that this might be one of the reasons for high tissue restricted expression in the placenta (45). The HLA-G suppression by miR-152 in JEG-3 cells, followed by increased susceptibility to NK cell-mediated cytolysis, has also been shown in a previous study (68). Soluble factors, like immune-modulatory hormones and cytokines have shown to influence the transcription of HLA-G. The rate of HLA-G transcription is increased by the cytokine IFN-β. The indoleamine 2,3-dioxygenase (IDO), which is an enzyme catabolizing tryptophan, has shown to increase the shedding and expression of HLA-G in myeloid dendritic cells (DCs), which contribute to a tolerogenic milieu (69). In fact, IDO has shown to promote maternal tolerance toward the fetus by catabolizing tryptophan and thereby suppressing the T cell activity in mice (70).

The acquirement of HLA-G by HLA-G-negative cells has been shown to be possible via trogocytosis. This mechanism is characterized by the transfer of surface molecules from one cell to another through cell–cell contact. Activated CD4^+^ and CD8^+^ T cells can acquire HLA-G from antigen-presenting cells (APCs) through this mechanism and thus contribute to an immune suppressive milieu without expressing HLA-G themselves, but only temporarily displaying it (71).

The trophoblast cells have an alternative HLA expression profile compared to all other cells in the human body, in that they only express the non-classical MHC class Ib (HLA-E, -F, and -G) molecules, and to some extend the classical MHC class Ia HLA-C. Usually, an altered HLA expression profile is associated with a pathological condition, such as a virus infection or in malignant transformation, and thus induces an immune response mediated by the engagement and cytolytic killing of the cell in question by NK cells. HLA-G, however, engages inhibitory molecules on leukocytes rendering them anergic toward the trophoblast cells, thereby protecting the allogeneic fetus (2, 65, 72). HLA-G interacts with the ILT-2 and ILT-4 receptors, the co-receptor CD8, and maybe the killer cell immunoglobulin-like receptor (KIR) 2DL4 (73).

### The HLA-G receptors

The uterine NK (uNK) cells, identified by being CD16^−^CD56^bright^, as opposed to peripheral CD16^+^CD56^+^ NK cells, express KIR2DL4, which is described as a receptor for HLA-G, although a recent study has made this controversial (74, 75). KIR2DL4 has an immunoreceptor tyrosine-based inhibitory motif (ITIM) at its cytoplasmic tail, and is characterized by its expression in endosomes. Despite having an ITIM, KIR2DL4 when possibly bound to HLA-G shows weak inhibition of uNK cell. Because of its endosomal expression, at least in steady state conditions, KIR2DL4 seems to bind sHLA-G, which may activate the uNK cell to secrete cytokines and chemokines important for angiogenesis (76). However, further studies are certainly needed to clarify the possible interactions between HLA-G and KIR2DL4.

Immunoglobulin-like transcript 2 and ILT-4 are inhibitory receptors expressed on leukocytes. These receptors also bind other HLA class I molecules, however, preferentially bind HLA-G (77). ILT-2 and ILT-4 contain three ITIMs at their cytoplasmic tail. ILT-2 (also named LILRB1) is expressed by monocytes, macrophages, CD4^+^ and CD8^+^ T cells, B cells, and myeloid DCs and engages only heterodimers of HLA-G1 or sHLA-G5 and β2m. ILT-4 (LILRB2) expressed by monocytes, macrophages, and myeloid DCs can also interact with HLA-G monomers. The discrepancy in HLA-G interaction with ILT-2 and ILT-4 has been clarified with the use of crystal structures showing that ILT-2 cannot recognize the β2m-free form of HLA-G, whereas ILT-4 preferably bind the α3 domain of the HLA-G heavy chain (78). In fact, by binding to its cognate inhibitory receptors, HLA-G has shown to up-regulate the expression of ILT-2 and ILT-4. The functional consequence of inhibitory receptor up-regulation by HLA-G was hypothesized to be an increased sensitivity toward inhibition mediated, not only by HLA-G, but also by classical HLA class I molecules known to bind ILT-2 and ILT-4 (79). The co-receptor CD8 expressed on cytotoxic T cells is also known to bind HLA-G. This interaction causes apoptosis of the activated CD8^+^ T cells mediated by the FAS ligand/FAS pathway (80).

## HLA-G in Reproductive Immunology

Cell–cell interactions between leukocytes and trophoblast cells mediated by HLA-G and its above-mentioned receptors are of great interest in order to understand the immune regulation at the feto-maternal interface (Figure 3). So far, several studies have tried to elucidate the strict immune regulation taking place at this anatomical site. A range of different studies indicate that HLA-G might have a central position in the immune regulation at conception and during pregnancy.

### NK cells, DCs, and T cells

It has become clear that DCs and T cells, especially Tregs, in the decidua are important contributors to the tolerogenic milieu in pregnancy and several studies have described such cells and their implications in healthy and in complicated pregnancies. Some of the described cell types have overlapping features. However, the NK cells are by far the most abundant cells in the uterus.

The early decidua is characterized by an abundance of uterine CD16^−/dim^CD56^bright^ NK cells that are in close contact with the fetal-derived extravillous trophoblast cells. uNK cells possess ILT-2, ILT-4, and KIR2DL4 receptors that bind HLA-G expressed on the surface of the infiltrating trophoblast cells. The CD16^−/dim^CD56^bright^ NK cells are the largest population of lymphocytes in the uterus, they constitute 50–90% of lymphocytes in human uterine decidua in early pregnancy. They are phenotypically and functionally distinct from conventional CD16^+^CD56^dim^ NK cells that circulate the periphery (81). They are recruited in large numbers through the first and second trimester and participate in the modification of the uterine spiral arteries, which increases blood flow to the fetus (82). The crosstalk between DCs and NK cells has been shown to be modulated by sHLA-G in cell cultures. DCs cultured with sHLA-G showed a reduced ability to induce NK cell activation (83). HLA-G non-amers have shown to be presented by HLA-E, which stabilizes the HLA-E molecule (84).

Gregori et al. have shown that a specialized tolerogenic type of DCs is accumulating in the human decidua during pregnancy. These cells express HLA-G and are named DC-10 because they secrete high amounts of IL-10. DC-10 can induce type 1 regulatory T (Tr1) cells, which are characterized by their cytokine profile – secretion of IL-10 and TGF-β among others (73, 85).

Thymic stromal lymphopoietin (TSLP) is expressed by the epithelial cells of Hassall’s corpuscles in the thymus and induce DCs to stimulate Treg differentiation. During pregnancy though, the function of the thymus is reduced, and the Treg expansion during pregnancy has been proposed to take place in the placenta since TSLP is produced by the trophoblast cells. The trophoblast cells are in close contact with DCs around the spiral arteries and TSLP likely activate the CD11^+^ DCs to secrete IL-10 and TGF-β and instructing them to induce the differentiation of immature T cells into CD4^+^CD25^+^FoxP3^+^ Tregs that also secrete IL-10 and TGF-β. The Tregs further induce the trophoblast cells to express HLA-G, which causes the decrease in uNK cytotoxicity. This tolerogenic loop was recently described by Du et al. (86). The DCs in the study by Du et al. secreted IL-10 as well as the HLA-G-expressing DC-10 described by Gregori et al. and it can be speculated if they might be part of the same DC subset. Also, HLA-G presenting CD4^+^ T cells have been identified at the feto-maternal interface, where they may contribute to the tolerogenic milieu (87).

In mice, it has been shown that the overall CD4^+^CD25^+^ suppressive T cell pool increases during pregnancy, that a third of the CD4^+^CD25^+^ T cells in the pregnant uterus express FoxP3, and that depletion of CD25^+^ T cells results in gestation failure (88).

## *HLA-G* Polymorphisms in Pre-eclampsia and Recurrent Miscarriages

Pre-eclampsia is a multisystemic pregnancy disorder that is manifested clinically in the late second and third trimester of pregnancy. The etiology of pre-eclampsia is unknown, although a substantial number of studies favor a theory based on a maladapted immune system, with the more specific attributes of low levels of immune regulatory cells and a low expression of HLA-G. The low expression is hypothesized partly to be a consequence of genetic variability. In support of this, a reduced level of *HLA-G* mRNA is observed in pre-eclamptic placentas, which directly correlates with the *HLA-G* genotype (5). Especially, the 14-bp ins/del 3′UTR polymorphism in exon 8 has been extensively studied, and this polymorphism has been found to be associated with severe pre-eclampsia in several studies (5, 6, 8, 89, 90). However, some studies have not observed an association between the 14-bp ins/del polymorphism and pre-eclampsia (91–93). Few studies address the issue that pre-eclampsia presents in a mild and a severe form, and furthermore, that it can be defined based on early- and late-onset, and importantly, that these forms potentially have distinct etiologies (94). Other polymorphisms associated with pre-eclampsia are typically present in the 5′URR and 3′UTR (8, 95).

*HLA-G* polymorphisms have been investigated in relation to recurrent miscarriages, also, with contradicting results (64, 96, 97). Two meta-analysis have addressed the possible association between the 14-bp ins/del and recurrent miscarriages: the first performed by Wang et al. including 14 studies, 1464 cases, and 1247 controls, found that the 14-bp ins/del is significantly associated with unexplained recurrent miscarriage, and suggests that the 14-bp-insertion increases the risk of recurrent miscarriage (98). These findings were challenged by another meta-analysis performed by Fan et al. including 17 studies, 1786 cases, and 1574 controls. The authors concluded that the body of evidence to demonstrate a conclusive association between the 14-bp ins/del with the risk of recurrent miscarriages is inadequate (99). In a subgroup analysis, however, they did find an association between the 14-bp ins/del polymorphism and risk of recurrent miscarriage in women, who suffered three or more miscarriages. Furthermore, they criticize the meta-analysis by Wang et al. for including two different studies based on the same study group (99).

An increasing amount of studies acknowledge that the etiology of various pregnancy complications is based on the unique immunogenetic combinations of the mother and the father. Paternal immunogenetic factors may indeed contribute to the risk of development of pre-eclampsia. One study shows that the paternal HLA-G G*01:06 contribution significantly increases risk for pre-eclampsia in multigravidas, who do not carry this allele (100).

### Regulatory immune cells in pre-eclampsia

Hsu et al. have recently published a study with the purpose of describing the role of immune regulatory cells in pre-eclamptic women (101). CD4^+^HLA-G^+^ T cells in the periphery and the decidua from healthy pregnant women, and from pre-eclamptic cases and non-pregnant women, were measured. In previous studies, APCs characterized by being CD14^+^DC-SIGN^+^HLA-G^+^ and ILT-4^+^ have been described. These could be the same DCs described by Gregori et al. (87). Like Tregs, this CD4^+^HLA-G^+^ T cell subset may play an important role in immune tolerance during pregnancy. In the periphery, increase of CD4^+^HLA-G^+^ T cells during healthy pregnancies was observed compared to non-pregnant controls. Pre-eclamptic women had a significantly lower fraction of CD4^+^HLA-G^+^ T cells than healthy pregnant women. CD4^+^HLA-G^+^ T cells seem to be more mature compared to CD4^+^HLA-G^−^ T cells because of their expression of CD80 and CD86. Also, the CD4^+^HLA-G^+^ T cells expand in the decidua compared to the periphery, but whether it is a local expansion of the T cell pool or recruitment from the periphery is not known. Again, pre-eclamptic women had a lower expansion of CD4^+^HLA-G^+^ T cells than healthy pregnant women. It was demonstrated that the CD4^+^HLA-G^+^ T cells acquire their HLA-G by trogocytosis. In this case from CD14^+^DC-SIGN^+^ DCs expressing HLA-G and ILT-4 (101).

## Future Aspects and Conclusion

As mentioned, HLA-G exists in a monomer form and a dimer form, the latter by forming an intermolecular disulfide bridge between two cysteine residues of the α1 domains of two HLA-G molecules (102). Studies indicate that the dimer is the most active form; it has a higher affinity than the monomer to ILT-2 and ILT-4, and the dimer enhances the ILT-2-mediated signaling at the cellular level (103). In line with this, focus has been drawn to HLA-G dimers and synthetic dimer HLA-G molecules for possible therapeutic use (104). In mice, recombinant sHLA-G and synthetic HLA-G molecules have been shown to inhibit the early stages of arthritis in a rheumatoid arthritis disease model and to significantly prolong the acceptance of skin grafts (104, 105). It can be speculated that synthetic sHLA-G analogs might find a place in the treatment of certain pregnancy-related disorders, such as pre-eclampsia and assisted reproduction. However, a better fundamental understanding of the pathophysiology in these disorders is needed before proceeding to such enterprises.

Also, in *in vitro* fertilization (IVF) treatments, the measurement of sHLA-G in the embryo culture medium can be used as a marker for improving successful assisted reproductive technology, by choosing the fertilized oocytes with highest potential, as sHLA-G positive culture medium correlates with pregnancy success (10, 58, 106).

In conclusion, for obtaining a successful conception and a pregnancy in terms of optimized immune modulation, a combined expression of HLA-G from several sources seems to be important: by the mother in the blood, in follicular fluid, and in the genital tract, by the embryo and the trophoblast cells in the placenta, and by the father through the presence of sHLA-G in semen. One of our recent studies even revealed a significant association between HLA-G genotype and the amount of sHLA-G in seminal plasma (16). More studies are needed to elucidate the precise roles and the importance of these different sources of HLA-G in relation to uncomplicated pregnancies and in pre-eclampsia, in recurrent miscarriage and in assisted reproduction, especially with respect to the control of HLA-G expression involving *HLA-G* gene polymorphisms together with molecular and cellular immune interactions.

## Conflict of Interest Statement

The authors declare that the research was conducted in the absence of any commercial or financial relationships that could be construed as a potential conflict of interest.
